# Long-term safety and efficacy of intramyocardial adenovirus-mediated VEGF-D^ΔNΔC^ gene therapy eight-year follow-up of phase I KAT301 study

**DOI:** 10.1038/s41434-021-00295-1

**Published:** 2021-10-01

**Authors:** Aleksi J. Leikas, Iiro Hassinen, Antti Hedman, Antti Kivelä, Seppo Ylä-Herttuala, Juha E. K. Hartikainen

**Affiliations:** 1 Heart Center, Kuopio University Hospital, Kuopio, Finland; 2 A.I. Virtanen Institute for Molecular Sciences, University of Eastern Finland, Kuopio, Finland; 3 Gene Therapy Unit, Kuopio University Hospital, Kuopio, Finland

**Keywords:** Cardiovascular diseases, Gene therapy

## Abstract

In phase I KAT301 trial, intramyocardial adenovirus-mediated vascular endothelial growth factor -D^ΔNΔC^ (AdVEGF-D) gene therapy (GT) resulted in a significant improvement in myocardial perfusion reserve and relieved symptoms in refractory angina patients at 1-year follow-up without major safety concerns. We investigated the long-term safety and efficacy of AdVEGF-D GT. 30 patients (24 in VEGF-D group and 6 blinded, randomized controls) were followed for 8.2 years (range 6.3–10.4 years). Patients were interviewed for the current severity of symptoms (Canadian Cardiovascular Society class, CCS) and perceived benefit from GT. Medical records were reviewed to assess the incidence of major cardiovascular adverse event (MACE) and other predefined safety endpoints. MACE occurred in 15 patients in VEGF-D group and in five patients in control group (21.5 vs. 24.9 per 100 patient-years; hazard ratio 0.97; 95% confidence interval 0.36–2.63; *P* = 0.95). Mortality and new-onset comorbidity were similar between the groups. Angina symptoms (CCS) were less severe compared to baseline in VEGF-D group (1.9 vs. 2.9; *P* = 0.006) but not in control group (2.2 vs. 2.6; *P* = 0.414). Our study indicates that intramyocardial AdVEGF-D GT is safe in the long-term. In addition, the relief of symptoms remained significant during the follow-up.

## Introduction

Despite continuous development of better methods for the treatment of coronary artery disease, there is still a significant number of patients suffering from angina pectoris in spite of the optimal, guidelines recommended pharmacological and invasive treatments. The prevalence of this sub-population, patients with refractory angina, has been estimated to represent 6–14% of all angina pectoris patients [1].

Gene therapy (GT) with members of the vascular endothelial growth factor (VEGF) family is a promising, investigational treatment option for alleviating symptoms of patients suffering from the refractory angina [2]. The aim of this treatment is to induce the formation of new blood vessels (angiogenesis) and vasodilation of the existing vessels (arteriogenesis) by increasing the expression of VEGFs [3, 4]. This can be achieved by genetically modified VEGF-encoding vectors, such as adenoviruses, which have the ability to induce angiogenesis and arteriogenesis when administered into the ischemic, but still viable myocardium [4]. So far, clinical studies have reported mixed results after adenovirus-mediated GT with different members and isoforms of the VEGF family [5–7].

Phase I KAT301 trial was the first study that investigated the safety and efficacy of intramyocardial adenovirus-mediated VEGF-D^ΔNΔC^ (AdVEGF-D) GT in patients with refractory angina [7]. The trial showed an increase in myocardial perfusion reserve in areas treated with AdVEGF-D injections when measured by ^15^O-H_2_O-postiron emission tomography. In addition, GT resulted in the improvement of angina pectoris symptoms and quality of life. Most importantly, the intervention was found well tolerated and safe with no serious adverse reactions linked to the GT product during the 1-year follow-up.

Although the results from the 1-year follow-up were encouraging, the long-term efficacy and safety of intramyocardial AdVEGF-D GT need to be established. Previously, we have reported the 8- and 10-year outcomes of patients treated with intracoronary and intramuscular adenovirus-mediated VEGF-A injections for coronary artery disease and peripheral artery disease [8, 9]. In these studies, the safety profile of angiogenic adenovirus-mediated GT was excellent. In the current study, our aim was to investigate whether the long-term safety of intramyocardial AdVEGF-D GT is comparable to the interventions in the previous clinical trials and whether the observed benefit in angina symptoms in KAT301 trial has persisted until today.

## Materials and methods

### Patient selection

The study population consisted of 30 patients who participated in the phase I KAT301 study (ClinicalTrials.gov NCT01002430) and suffered from refractory angina pectoris despite optimal medical therapy. Recruited patients were not eligible for revascularization, i.e., percutaneous coronary interventions (PCIs) or coronary artery bypass grafting. The patients were randomized to undergo either intramyocardial AdVEGF-D GT (VEGF-D group) or sham procedure (control group) in 4:1 ratio between years 2010–2014. The patients were blinded throughout the 1-year follow-up, after which the blinding was opened. The baseline characteristics of the study population are presented in Table 1.

**Table 1:** Baseline clinical characteristics of the study groups.

	VEGF-D (*n* = 24)	Control (*n* = 6)	*P* value
Demographics			
Sex (male)	23 (96)	5 (83)	0.67
Age (years)	71 (68.6–73.4)	70 (65.2–74.8)	0.40
CCS-class	2.8 (2.7–3.0)	2.7 (2.3–3.1)	0.56
Medical history			
Previous MI	17 (71)	4 (67)	0.60
Previous CABG	23 (96)	6 (100)	0.80
Previous PCI	15 (63)	3 (50)	0.46
Family history of CAD	19 (79)	5 (83)	0.66
Hypertension	22 (92)	6 (100)	0.63
Hypercholesterolaemia	23 (96)	6 (100)	0.80
Smoker (current/ex)	0/17 (0/71)	0/4 (0/67)	0.90
Diabetes	13 (54)	3 (50)	1.00
Medication			
Aspirin	22 (92)	5 (83)	0.51
Clopidogrel	12 (50)	3 (50)	1.00
Warfarin	8 (33)	2 (33)	1.00
B-blockers	24 (100)	6 (100)	1.00
ACE-I/ARB	21 (88)	6 (100)	0.49
Statins	24 (100)	5 (83)	0.20
Long-acting nitrates	23 (96)	5 (83)	0.37

The values are mean (95% confidence intervals) or *n* (%).

*ACE-I* angiotensin converting enzyme inhibitors, *ARB* angiotensin receptor blockers, *CAD* coronary artery disease, *CABG* coronary artery bypass grafting, *CCS* Canadian Cardiovascular Society, *MI* myocardial infarction, *PCI* percutaneous coronary intervention, *VEGF-D* vascular endothelial growth factor D.

### Gene therapy

VEGF-D^ΔNΔC^ encoding first-generation serotype 5 adenoviruses with a deletion in E1 and partial deletion in E3 genomic regions were produced under Good Manufacturing Practice by FinVector Oy (Kuopio, Finland). The product was administered as 10 intramyocardial injections (200 μL each) using NOGA*®* electroanatomical mapping system with MyoStar*®* injection catheter (Johnson & Johnson, Diamond Bar, California, USA) into the areas of viable myocardium with decreased myocardial perfusion reserve [10]. For the control group, the electromechanical mapping was conducted in the same way as in the VEGF group, but instead of intramyocardial AdVEGF-D injections, 10 NaCl boluses (200 uL each) were administered into the left ventricle.

### Follow-up

The average follow-up period was 8.2 years (range 6.3–10.4 years). Patients were interviewed by letter and by phone using a questionnaire modified from our previous studies [8, 9] containing standardized questions about their post-interventional medical history, severity of symptoms (Canadian Cardiovascular Society class, CCS) and perceived benefit from the GT (positive, no change, negative, and unable to answer). The interviews were conducted in a one-time fashion during the spring 2020. After the interviews, the patient medical records were reviewed in combination with the answers to the questionnaires.

The safety of GT was assessed by evaluating the incidence of major cardiovascular adverse event (MACE), a composite endpoint of acute coronary syndrome, PCI, coronary artery bypass grafting, stroke, and cardiovascular death. Also, the individual components of MACE were assessed. In addition, the incidence of clinical arrhythmias (defined as an arrhythmia diagnosed by 12-lead electrocardiogram and resulting in drug or device therapy), malignancies, diabetes [11], proliferative retinopathy, and chronic kidney disease with a severely decreased estimated glomerular filtration rate [12] (eGFR < 30 mL/min/1.73 m^2^, using Chronic Kidney Disease Epidemiology Collaboration equation) were recorded. For the patients that had deceased during the follow-up, the causes of death were reviewed from the national death register (Statistics Finland). All patients gave their informed consent, and the study protocol was approved by Research Ethics Committee of the Northern Savo Hospital District. The study was conducted in accordance with the Declaration of Helsinki and following local laws concerning pharmaceutical interventional trials.

### Statistical methods

Logrank test was used for the analysis of MACE incidence and hazard ratio with 95% confidence interval (CI) was calculated. CCS class was analyzed using two-sided Wilcoxon signed-rank test and presented as mean and 95% CI assuming *t*-distribution. Two-sided Fisher’s exact test was used for dichotomous variables. Statistical significance was ascribed at *P* value < 0.05. All statistical analyses were conducted using GraphPad Prism version 9 (GraphPad Software, La Jolla, California, USA) and SPSS Statistics version 27 (IBM, Armonk, New York, USA).

## Results

### Major adverse cardiovascular events

**Fig. 1:**
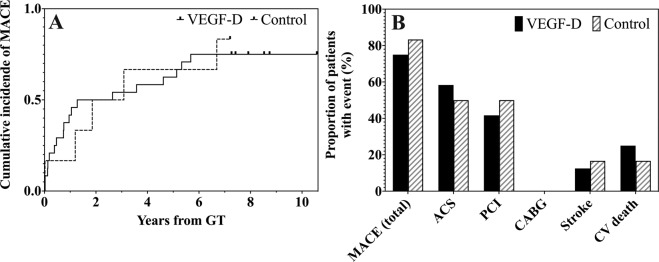
Incidence of major adverse cardiovascular event (MACE). **A** The solid line represents the cumulative incidence of MACE in the VEGF-D group and the dashed line represents the incidence in the control group. **B** The bars show the proportion of patients with MACE or its individual component during the follow-up. ACS acute coronary syndrome, CABG coronary artery bypass grafting, CV cardiovascular, GT gene therapy, MACE major adverse cardiovascular event, PCI percutaneous coronary intervention, VEGF-D vascular endothelial growth factor D.

A MACE occurred in 18 (18/24, 75.0%) patients in the VEGF-D group (21.5 per 100 patient-years) and in five (5/6, 83.3%) patients in the control group (24.9 per 100 patient-years) with no difference in the incidence between the groups (hazard ratio in the VEGF-D group, 0.97; 95% CI, 0.36–2.63; *P* = 0.95) (Fig. 1a). In addition, the observed differences with respect to the individual MACE components were not significant between the VEGF-D and control groups (Fig. 1b).

Seeing that nearly half of the patients had undergone PCI, it was interesting to evaluate the indications for these interventions, as the inclusion criteria for KAT301 trial was a non-eligibility for the invasive coronary procedures. Reinterventions were performed to treat the symptoms caused either by a de novo lesion, i.e., lesion not present at the time of screening or to recanalize a chronic total occlusion using advanced PCI techniques that became available after KAT301. De novo lesion was treated in seven patients (7/24, 29.2%) in the VEGF-D group (5.9 per 100 patient-years) and in two patients (2/6, 33.3%) in the control group (6.3 per 100 patient-years) (*P* = 1.00), whereas chronic total occlusion recanalization was performed to three patients (3/24, 12.5%) in the VEGF-D group (2.0 per 100 patient-years) and to one patient (1/6, 12.7%) in the control group (2.6 per 100 patient-years) *P* = 1.00). These interventions were spread evenly throughout the course of the follow-up in both groups.

### Mortality

During the follow-up, eight (8/24, 33.3%) patients deceased in the VEGF-D group (5.3 per 100 patient-years) and one (1/6, 16.7%) patient in the control group (2.7 per 100 patient-years) with no observed difference between the VEGF-D and control groups (*P* = 0.64) (Table 2). Seven deaths were cardiac deaths (7/9, 77.8%), consisting of two sudden cardiac deaths, and one that was due to exacerbation of congestive heart failure. Rest of the cardiac deaths were caused by a myocardial infarction. A hepatic tumor was described as the cause of death in two patients (2/9, 22.2%), both of which belonged to the VEGF-D group.

**Table 2:** Mortality and causes of death.

	VEGF-D (*n* = 24)	Control (*n* = 6)	*P* value
Overall mortality	8/24 (33.3) [5.3]	1/6 (16.7) [2.7]	0.64
Cardiac death	6/8 (75.0) [4.0]	1/1 (100.0) [3.4]	1.00
Malignancy	2/8 (25.0) [1.3]	0/8 (0.0) [0.0]	1.00

*VEGF-D* vascular endothelial growth factor D.

The values denote *n* (%) and [per 100 patient-years]. The overall mortality is presented as *n* (%) and [per 100 patient-years]. For the causes of death, the values denote *n* (total %) per deceased patients and [per 100 patient-years]. Fisher’s exact test.

### New-onset comorbidities

In total, new malignancies were diagnosed in six (6/24, 25.0%) patients in the VEGF-D group (4.2 per 100 patient-years) and one (1/6, 16.7%) patient in the control group (2.4 per 100 patient-years) with the observed incidence showing no difference between the groups (*P* = 1.00) (Table 3). The diagnoses included small lymphocytic lymphoma, two prostate cancers, basal cell carcinoma, chromophobe renal cell carcinoma, hepatocellular carcinoma, myelodysplastic syndrome, and an unspecified tumor of the liver with no biopsy taken. One patient had both prostate cancer and basal cell carcinoma.

**Table 3:** Incidence of new-onset comorbidities.

	VEGF-D (*n* = 24)	Control (*n* = 6)	*P* value
Malignancy	6/24 (25.0) [4.2]	1/6 (16.7) [2.4]	1.00
Arrhythmia	7/14* (50.0) [8.3]	3/4* (75.0) [12.9]	0.59
Type 2 diabetes	3/11^#^ (27.3) [3.7]	0/3^#^ (0.0) [0.0]	1.00
Proliferative retinopathy	0/24 (0.0) [0.0]	0/6 (0.0) [0.0]	NA
Chronic kidney disease	4/24 (16.7) [2.9]	1/6 (16.7) [3.3]	1.00

*VEGF-D* vascular endothelial growth factor D.

The values denote *n* (%) and [per 100 patient-years]. Fisher’s exact test. Chronic kidney disease = estimated glomerular filtration rate <30 mL/min/1.73 m^2^. Number of patients without * = arrhythmias and ^#^ = type 2 diabetes at baseline.

New clinical arrhythmias were encountered in seven (7/14, 50.0%) patients in the VEGF-D group (8.3 per 100 patient-years) and in three (3/4, 75.0%) patients in the control group (12.9 per 100 patient-years) (*P* = 0.59). New-onset type 2 diabetes was diagnosed in three (3/11, 27.3%) patients, all of which belonged to the VEGF-D group (3.7 per 100 patient-years) (*P* = 1.00). Proliferative diabetic retinopathy was not found in any of the patients. During the screening, three (3/24, 12.5%) patients in the VEGF-D group and two (2/6, 33.3%) patients in the control group presented with reduced kidney function (eGFR < 45 mL/min/1.73 m^2^); further development of chronic kidney disease with a severely decreased eGFR (<30 mL/min/1.73 m^2^) was encountered in four (4/24, 16.7%) patients in the VEGF-D group (2.9 per 100 patient-years) and one (1/6, 16.7%) patient in the control group (3.3 per 100 patient-years) (*P* = 1.00).

### Angina pectoris and perceived benefit from gene therapy

**Fig. 2:**
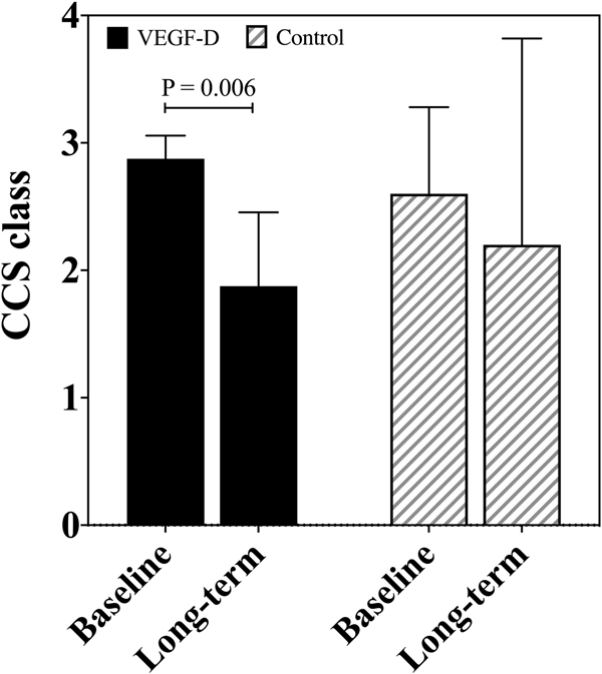
Canadian Cardiovascular Society (CCS) score at baseline and after the follow-up. The bars represent mean with 95% confidence interval. CCS Canadian Cardiovascular Society, VEGF-D vascular endothelial growth factor D.

At the time of the interview, seven (7/16, 43.8%) patients in the VEGF group and one (1/5, 20.0%) patient in the control group reported no chest pain or equivalent symptoms. In addition, patients in the VEGF group reported less severe angina symptoms (CCS) compared to the baseline (1.9; 95% CI 1.3–2.5 vs. 2.9; 95% CI 2.7–3.1; *P* = 0.006), but there was also a trend of less severe symptoms in the controls (2.2; 95% CI 0.6–3.8 vs. 2.6; 95% CI 1.9–3.3; *P* = 0.414) (Fig. 2).

In general, patients in both groups reported a benefit from the intervention (12/16, 75.0% vs. 2/5, 40.0% in the VEGF-D and control groups, respectively) (Table 4). None of the patients reported worsening of their symptoms.

**Table 4:** Perceived benefit.

	VEGF-D (*n* = 16)	Control (*n* = 5)	*P* value
Positive	12 (75.0)	2 (40.0)	0.28
No change	1 (6.3)	2 (40.0)	0.13
Negative	0 (0)	0 (0)	NA
Unable to answer	3 (18.8)	1 (20.0)	1.00

*VEGF-D* vascular endothelial growth factor D.

The values denote *n* (%). Fisher’s exact test.

## Discussion

Our key finding was that during the 8-year follow-up refractory angina carries a high risk of cardiovascular events, particularly reinterventions, and high cardiovascular mortality. However, we found no evidence of safety concerns related to AdVEGF-D GT.

Although the results from the registry-based study conducted on OPTIMIST database consisting a total of 1200 refractory angina patients indicate that the mortality in this patient population may be lower (one year 3.9%, nine years 28.4%) than previously suspected [13], the rate of hospitalization due to recurrent cardiovascular events still remains high [14, 15]. The mortality in our study (2.7–5.3 deaths per 100 patient-years) was in line with OPTIMIST database. In addition, it was not a surprise for us that only a minority of KAT301 patients had survived without encountering some components of MACE regardless of the study group.

A safety concern is that iatrogenic upregulation of VEGFs may result in the progression of a subtle cancer by various signaling pathways [16]. Although the incidence of malignancies did not differ in the VEGF-D and control groups, we were somewhat surprised to find that two patients in the VEGF-D group developed a hepatic cancer during the follow-up period. In both of these patients, the cancer was found incidentally in an ultrasound examination two and six years after the GT. A liver biopsy was taken from another patient and the subsequent histopathological examination revealed the cancer to be hepatocellular carcinoma. The patient was treated with sorafenib in combination with palliative radiation therapy to the spine metastases. However, as the cancer continued to progress despite the oncological therapies, the patient was eventually shifted to receive terminal phase care. In the second patient, it was not possible to attain a biopsy from the tumor due to the patient’s frail overall condition, and the treatment strategy was quickly chosen to be palliative rather than curative. Both patients deceased in less than a year from the initial diagnosis. Because there was no clinical uncertainty in the causes of death, autopsies were not conducted, and hence no additional testing was done to assess the presence of vector DNA.

It has been shown that current administration techniques for adenoviral vectors cause systemic off-target distribution [17], and that the main organ responsible for the vector elimination is the liver [18]. Even though the time interval between GT and the diagnosis of cancer was two years or more, this concern needs to be carefully monitored in the future trials. In our previous studies we have not observed anything that would raise concern on the potential risk of cancer [7–10]. However, it is of paramount importance that any patients with history of cancer should be excluded from the future AdVEGF-D trials and the recruited subjects should be strictly monitored throughout the study.

Angiogenic growth factors can potentially induce proliferative retinopathy and diabetic nephropathy [19]. It has also been suggested that VEGFs play a role in the development of arteriovenous malformations [20], and thus increase the risk of hemorrhagic strokes. In addition, there might be a risk of arrhythmogenicity related either to the gene transfer or the administration technique [21, 22]. Fortunately, we did not observe any cases of proliferative retinopathy in our patients, and the incidences of arrhythmias, hemorrhagic strokes, and severe chronic kidney disease were similar between the groups. This is in line with our previous results from the long-term follow-up of patients treated with intracoronary or intramuscular administration of adenovirus-mediated VEGF-A GT in coronary artery disease or peripheral artery disease [8, 9].

As KAT301 was a phase I trial, its sample size was chosen primarily to fulfill the purpose of evaluating the feasibility of AdVEGF-D GT. Because of this, although the statistical analysis showed no group differences in the mortality or safety outcomes, e.g., the incidence of cancer, it is important to be aware that this might had been simply because the groups were different in their size and the overall study population was relatively small (VEGF-D *n* = 24; control *n* = 6). We think that additional studies are needed in the future before any definitive conclusions on the long-term safety of the intervention can be made.

Contrary to our previous studies, we found that the benefit from GT had persisted after the initial follow-up. However, we would like to point out that this result must be interpreted with caution, as there was a major difference in the group sizes, the overall sample size was small, and almost half of the patients had undergone PCI during the follow-up. There is a possibility that the relief in angina symptoms had resulted not only from GT but also from the coronary interventions, but the statistical power to present this was sufficient only in the VEGF-D group. In addition, the patients were aware of their treatment group at the time of the interviews, which might have had a significant influence on their perception of symptoms.

## Conclusions

Our results suggest that the safety profile of intramyocardial AdVEGF-D GT is comparable to the placebo treatment within the 8-year follow-up. In addition, our results suggest that the treatment might relieve angina symptoms in the long-term. Because of the limitations in the study population, future studies are needed to confirm these findings.
